# Elevated Plasma Soluble Triggering Receptor Expressed on Myeloid Cells-1 Level in Patients with Acute Coronary Syndrome (ACS): A Biomarker of Disease Severity and Outcome

**DOI:** 10.1155/2021/8872686

**Published:** 2021-03-15

**Authors:** Shachaf Shiber, Vitaly Kliminski, Katia Orvin, Iftach Sagy, Mordehay Vaturi, Ran Kornowski, Michael Drescher, Yair Molad

**Affiliations:** ^1^Institute of Rheumatology, Rabin Medical Center, Beilinson Campus, Petach Tikva, Israel; ^2^Emergency Department, Rabin Medical Center, Beilinson Campus, Petach Tikva, Israel; ^3^Sackler Faculty of Medicine, Tel Aviv University, Tel Aviv, Israel; ^4^Laboratory of Inflammation Research, Felsenstein Medical Research, Petach Tikva, Israel; ^5^Cardiology Department, Rabin Medical Center, Beilinson Campus, Petach Tikva, Israel; ^6^Clinical Research Center, Soroka University Medical Center, Beer Sheva, Israel

## Abstract

**Background and Aims:**

Plasma levels of soluble triggering receptor expressed on myeloid cells (sTREM-1) reflect innate immune cell activation. We sought to evaluate sTREM-1 levels in patients with acute coronary syndrome (ACS) and their predictive value for disease severity and outcome.

**Methods:**

Plasma sTREM-1 levels were prospectively measured by ELISA in 121 consecutive patients with new-onset (≤24 h) chest pain at arrival to the emergency department (ED) and 73 healthy controls. Secondary endpoints were the association of plasma levels of sTREM-1 with day 30 and month 6 major adverse cardiovascular events (MACE) defined as death, ACS, stroke, and need for coronary revascularization, as well as with CAD severity. The primary endpoint of the study was the association of plasma sTREM-1 level at the time of admission to the ED with a diagnosis of ACS at day 30.

**Results:**

Fifty-nine patients (48.7%) were diagnosed with ACS and 62 (51.3%) with nonspecific chest pain (NSCP). Median plasma sTREM-1 level at admission was significantly higher in the ACS group than the NSCP group and the control group (539.4 ± 330.3 pg/ml vs. 432.5 ± 196.4 pg/ml vs. 230.1 ± 85.5 pg/ml, respectively; *P* < 0.001) and positively correlated with the number of stenosed/occluded coronary arteries on angiography (*P* < 0.001). On logistic regression analysis, higher sTREM-1 levels predicted definite ACS vs. NSCP determined on day 30 (OR 1.29, 95% CI 1.07-1.54, *P* = 0.01) as well as with recurrent ACS (*P* = 0.04) and stroke (*P* = 0.02) at 6 months.

**Conclusions:**

Plasma sTREM-1 levels are significantly elevated in patients with ACS and might serve as a biomarker differentiating ACS from NSCP in the ED as well as an inflammatory biomarker for coronary artery disease severity and outcome.

## 1. Background

Coronary artery disease (CAD) is characterized by atherosclerosis of the vessels of the heart and commonly leads to morbidity and mortality [1]. The vast majority of cardiovascular events are caused by rupture or erosion of atherosclerotic plaque along an arterial wall with subsequent formation of an occluding thrombus. However, more than merely an obstructive vascular disease, CAD involves chronic low-grade vascular inflammation that contributes to atherogenesis [2–4].

Macrophages are the most abundant inflammatory cells in atherosclerotic plaque. Monocytes and macrophages apparently play a pivotal role in the initiation, progression, and destabilization of atherosclerotic plaque by several mechanisms, including necrosis and subsequent release of proinflammatory factors [5]. However, the exact processes that drive the persistent nonresolving inflammation in the vessel wall in atherogenesis remain incompletely understood. It has been suggested that macrophage activation is mediated by toll-like receptor- (TLR-) 4 and TLR-6 heterodimers [5, 6] that assemble in response to aggregation of oxidized low-density lipoprotein (ox-LDL) within the atherosclerotic plaque [7].

ox-LDL has also been found to play a role in the upregulation of triggering receptor expressed on myeloid cells- (TREM-) 1 (CD354) [8], an inflammatory receptor that is expressed on the surface of neutrophils and monocytes/macrophages. TREM-1 interacts with TLR-4 to amplify the proinflammatory response [9, 10]. Upon membrane TREM-1 activation, a soluble form of TREM-1 (sTREM-1) is cleaved in a metalloproteinase- (MMP-) 9-mediated process and shed to the circulation where it has been shown to exert an anti-inflammatory effect [11, 12]. The TREM-1-mediated proinflammatory effect has been identified in various infectious and noninfectious inflammatory conditions [9] including atherosclerosis [13].

Moreover, elevated levels of plasma sTREM-1 were found in patients with acute myocardial infarction (MI) and correlated with a higher risk of death [14]. Acute coronary syndrome (ACS) is a group of clinical conditions that include myocardial infarction (MI) with or without elevation of the ST segment on electrocardiogram (ECG) and unstable angina. Acute MI is classified according to electrocardiographic changes, such as non-ST elevation MI (NSTEMI) and ST elevation MI (STEMI). The prerequisite for diagnosis of acute MI is evidence of elevated serum high sensitivity cardiac troponin (hs-cTn) level that is released from injured cardiac myocytes, and its assay produces analytically reliable results at the 99th percentile of a healthy population which may facilitate identification of patients suitable for discharge to outpatient care. However, a second measurement of hs-cTn from a blood sample drawn two to four hours after hospital attendance time point is frequently required to confirm the diagnosis of ACS [15]. Although the main clinical symptom of ACS is chest pain, as few as 10% of patients with chest pain are ultimately diagnosed with ACS [16]. Thus, early diagnosis of ACS in patients who present to the ED with chest pain remains a diagnostic challenge for ED physicians, and there is an unmet need for novel strategies to identify low-risk patients at presentation to reduce hospital admissions as well as to improve care for patients. Blood biomarkers reflecting the activity of biological processes involved in atherosclerotic plaque growth and destabilization may serve as a clinical tool to reach an accurate diagnosis and determine the appropriate management for patients who present to the ED with a complaint of chest pain.

Given that TREM-1 is apparently involved in plaque destabilization and is upregulated in symptomatic atherosclerotic vascular disease [6–8], we hypothesized that plasma sTREM-1 might serve as a useful biomarker for discriminating ACS from non-ACS chest pain and vascular inflammation activity. The aim of this study was to evaluate levels of plasma sTREM-1 in the early phase of ACS and to determine its predictive value for day 30 and month 6 cardiovascular outcome.

## 2. Methods

### 2.1. Design and Setting

A prospective case-control design was used. The study was conducted in the emergency department (ED) of a tertiary university-affiliated medical center from March 2017 to March 2019. The protocol was approved by the Institutional Review Board of Rabin Medical Center (149-017–RMC), and all participants (patients and healthy control subjects) signed an informed consent form.

### 2.2. Sample Size

Studies have shown that 10% of patients who present to the ED with a complaint of chest pain are diagnosed with ACS [16]. Therefore, to establish a cohort of 100 patients with ACS, we screened 1000 consecutive patients presenting to the ED who met the study criteria. ACS was defined as a diagnosis of ST-segment elevation myocardial infarction (STEMI), non-ST-segment elevation myocardial infarction (NSTEMI), or unstable angina (UA) [17]. Patients in whom ACS was ruled out were diagnosed with nonspecific chest pain (NSCP). For the purpose of evaluating the ability of sTREM-1 levels to discriminate between patients with ACS or NSCP, we randomly selected a control group out of the 900 patients screened in the ED during the same period in whom ACS was ruled out. For the purpose of analyzing outcome, patients were divided into ACS and NSCP groups according to their definite diagnosis on day 30 of the study follow-up period. Individuals with no evidence of an inflammatory, malignant, and/or cardiac disease were served as the healthy control (HC) group.

### 2.3. Study Cohort: Inclusion/Exclusion Criteria

Inclusion criteria for the study group were consecutive patients, male or female, aged 18-80 years who presented to the ED between March 2017 and March 2019 with a chief complaint of chest pain suggestive of ACS that had started within 24 hours prior to admission. We excluded patients who had had an infectious disease within 90 days before onset of the chest pain, patients who had a malignant disease (except skin basal cell carcinoma) with/without chemo-/immuno-/radiotherapy within 12 months prior to the time of enrolment, pregnant women or women who had given birth or had a fetal loss within 3 months of onset of the chest pain, patients with any known autoimmune and/or inflammatory systemic disease, and patients who sustained chest wall trauma within 30 days of onset of the chest pain.

### 2.4. Study Endpoints

The primary endpoint of the study was the association of plasma sTREM-1 level at the time of admission to the ED with a diagnosis of ACS at day 30. Secondary endpoints were the association of plasma levels of sTREM-1 with day 30 and month 6 major adverse cardiovascular events (MACE) defined as death, MI (STEMI/NSTEMI), stroke, and need for coronary revascularization, as well as with CAD severity, recurrent hospital admission due to CAD, and the association with serum troponin-T levels, white blood cell count, and serum high-sensitive C-reactive protein (hsCRP) level.

### 2.5. Study Protocol

#### 2.5.1. Data Collection

Demographic and clinical data were systematically collected by patient interview at the time of enrolment by one of the study investigators (S.S. or KO) and retrieved into a central database. Patients underwent physical examination, and ECG, and blood was sampled for measurement of plasma sTREM-1 levels. Angiography was performed in-hospital or during follow-up. Clinical follow-up information about the endpoints was obtained after 30 days and after 6 months by reviewing the hospital database and by telephone calls to all patients or their families and then verified by reviewing the medical records.

#### 2.5.2. Patient Evaluation

At enrolment, data were obtained on age, sex, evidence of previous IHD, hypertension, diabetes mellitus, dyslipidemia, smoking (ever/current, pack-years), family history of IHD, and body mass index. Patients were examined for signs of heart failure and blood pressure, and blood samples were sent to the hospital's laboratory for measurement of white blood cell count and levels of serum creatinine, hsCRP, and serum creatinine phosphokinase (CPK). High-sensitivity cardiac troponin level (hs-cTnT) was measured with a highly sensitive assay (troponin T hs Stat; Roche Diagnostics, Indianapolis, IN, USA). According to the manufacturer, the coefficient of <10% is 13 ng/l and the 99th percentile of a healthy reference population is <13 ng/l. Therefore, all results equal to or below the 99th percentile are reported by the laboratory as a value of <13 ng/ml. In addition, all patients underwent serial ECG, and the findings were categorized as follows: normal, nonspecific T-wave changes, presence and location of ST-segment depression ≤ 1 mm, presence and location of ST-segment elevation ≥ 1 mm, and presence of q-wave.

On receipt of the clinical, laboratory, and ECG results at the ED, a decision was made to either admit the patient to the cardiac intensive care unit or an internal medicine ward or to discharge the patient if an ACS event was ruled out. For purposes of the study, patients were categorized as having ACS (STEMI, NSTEMI, unstable angina, and advanced angina pectoris) or NSCP.

Coronary artery angiography was performed according to clinical indications, and the result was recorded as one of the following: normal, coronary artery irregularity with no evidence of stenosis or occlusion, and single/double/triple-vessel coronary disease. Percutaneous coronary intervention (PCI) and/or aortocoronary bypass surgery (CABG) were performed at the discretion of the cardiologist based on guidelines/recommended practice.

The outcome of the ACS and NSCP groups was determined on day 30 and after 6 months of the study period. Outcomes were categorized as death, need for coronary revascularization (PCI and/or CABG), heart failure (defined as left ventricular dysfunction demonstrated on echocardiography along with clinical signs), stroke, and/or recurrent admission to the hospital due to ACS.

#### 2.5.3. Measurement of sTREM-1

Venous blood (10 cc) was drawn into vacutainer tubes containing heparin at the time of the first routine blood tests at admission to the ED and immediately centrifuged. The plasma samples were kept frozen at -80°C until assayed. The plasma sTREM-1 level was analyzed by a commercial ELISA kit (Human TREM-1 DuoSet ELISA kit, DY1278B, Bio-Techne, Minneapolis, MN, USA) according to the manufacturer's instructions.

#### 2.5.4. Statistical Analysis

The statistical analysis was generated using SPSS software, version 25.0. Data are expressed as mean and standard deviation (SD), median and interquartile range (IQR), or number and percentage. Patient characteristics were compared between groups using analysis of variance, chi-square test, and Kruskal-Wallis test. Correlations (*r*) were calculated by Pearson correlation. To estimate the association of the dependent variable with the development of ACS vs. NSCP, we conducted a forward stepwise conditional logistic regression. Each set of covariates (demographic, medical history, laboratory, etc.) was entered as a separate block into the model. The final model was selected based on goodness of fit using the c-statistic and plausible clinical explanation. We used the same method to analyze covariates that were associated with ACS vs. NSCP with inclusion of the log of plasma sTREM-1 levels.

Hundred divided plasma sTREM-1 levels in order to achieve a more convenient odds ratio (OR). Two-sided *P* values less than 0.05 were considered statistically significant.

## 3. Results

### 3.1. Patient Characteristics

**Figure 1:**
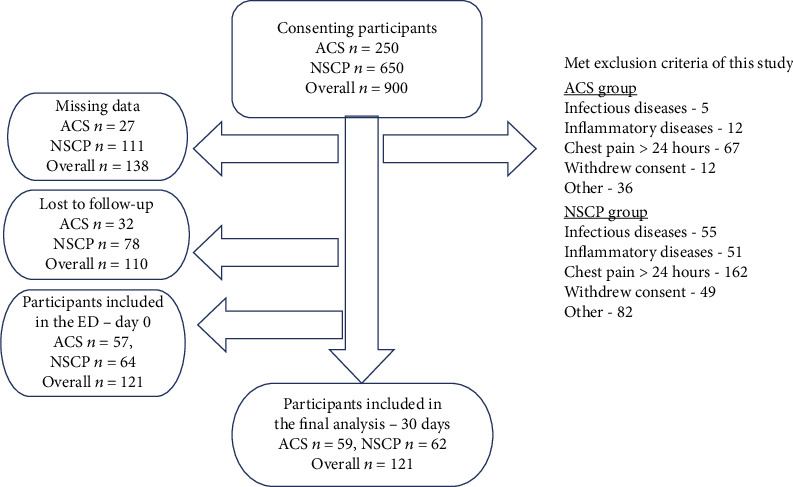
Flow chart of *n* = 900 patients presenting to the ED with suspected ACS.

Nine-hundred patients with suspected ACS were evaluated from March 2017 to March 2019, of whom 779 were excluded (Figure 1). The final study cohort consisted of 121 patients: 59 (48.7%) with a definite diagnosis of ACS as determined on day 30 and 62 (51.3%) with a definite diagnosis of NSCP. Within the ACS group, STEMI was diagnosed in 22 patients (37.3%), NSTEMI in 8 (13.6), and unstable angina in 29 (49.2%). The control group (HC) consisted of 73 healthy subjects. The background characteristics and the laboratory parameters of the study participants are depicted in Table 1. Compared to the NSCP group, the ACS group had a significantly higher mean age and had accrued significantly more risk factors for CAD, such as hypertension, dyslipidemia, smoking history, and diabetes mellitus. The ACS group also had a significantly higher serum troponin-T level than the NSCP group (*P* < 0.001). The WBC was significantly higher in the ACS than the NSCP group (*P* = 0.001), but there was no significant between-group difference in the serum hsCRP level.

### 3.2. Clinical Diagnosis

Of the 59 patients with a definite diagnosis of ACS, two were initially discharged from the ED with a diagnosis of NSCP. The diagnosis of ACS was made following their readmission during the 30 days following admission to the ED (Table 2). Fifty-nine patients (96%) underwent coronary angiography, and 53 underwent PCI, either at the time of hospitalization (*n* = 38, 64.4%) or during the 30-day follow-up period (*n* = 15, 29.5%). The findings are depicted in Table 3. The left anterior descending artery (LAD) and right coronary artery (RCA) were the vessels most frequently affected with either severe stenosis or occlusion (26 patients, 44.1% each). A median of two coronary arteries per patient (IQR 1-5) had lumen stenosis of >50%; mean diameter of the stenosis was 79 ± 15%. CABG was performed in four patients (6.8%) in the ACS group. None of the patients died during the 6 months follow-up period of the study.

### 3.3. Plasma sTREM-1 Level Is Elevated in Patients with ACS

**Figure 2:**
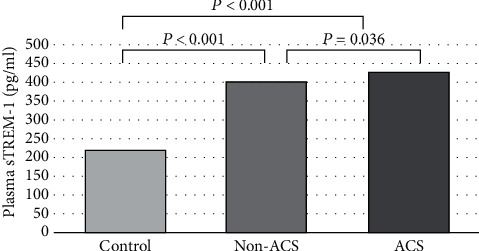
Median plasma levels of soluble TREM-1 in acute coronary syndrome compared to nonspecific chest pain and healthy control groups (using univariate logistic regression).

The median plasma sTREM-1 level was significantly higher in the ACS group compared to the HC group, 429.4 (281.7-762.9) pg/ml vs. 218.9 (181.7-277.0) pg/ml, *P* < 0.001. The median plasma level of sTREM-1 in the NSCP group (402.2 (302.6-572.2) pg/ml) was as well significantly higher than the HC group (*P* < 0.001). On logistic regression analysis, the plasma level of ACS groups was significantly higher than the NSCP group (*P* = 0.036) (Figure 2). Of note, the hrCRP level did not correlate with ACS or differentiated between patients with NSCP vs. ACS (*P* = 0.4, Table 2).

Owing to the wide range of plasma sTREM-1 levels found in healthy individuals in previous studies, the normal cutoff has not been determined.

Thus, in the present study, we used the 99th percentile of plasma sTREM-1 level in the healthy control group (median: 423 pg/ml) as the reference value.

We found that of the entire cohort of 121 patients presenting with chest pain, 60 (49.6%) had a median plasma sTREM-1 level above the 99th percentile of the healthy controls. This group had a significantly higher rate of coronary artery stenosis/occlusion on angiography than patients with sTREM-1 below the 99th percentile (*P* = 0.001), and a higher rate of and stroke on day 30 (*P* = 0.02), as well as MI (*P* = 0.04) and stroke (*P* = 0.02) at month 6. However, there was no significant between-group difference in the risk of MACE at day 30 and month 6 (Table 3). On logistic regression analysis, higher plasma sTREM-1 levels predicted ACS vs. NSCP and HC (OR 1.003, 95% CI 1.001–1.004, *P* = 0.01) (Table 4) and ACS vs. NSCP in patients presenting to the ED with chest pain (OR 1.29, 95% CI 1.07-1.54, *P* = 0.01) (Table 5).

When plasma levels of sTREM-1 in different ACS subgroups (STEMI, NSTEMI, and unstable angina) were analyzed using Bonferroni correction for multiple comparisons, the results did not reach a statistical significance.

### 3.4. Plasma sTREM-1 Level Correlates with CAD Severity

**Figure 3:**
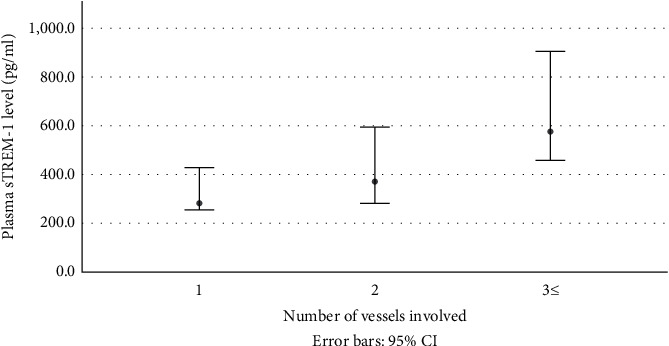
Median (i/q range) of plasma sTREM-1 level (pg/ml) in ACS patients stratified by number of stenosed/occluded coronary arteries as diagnosed on cardiac catheterization (*P* < 0.001).

Whereas serum troponin-T level and hrCRP did not correlate with the number of stenosed and/or occluded coronary arteries on the angiogram (the median (range) troponin-T level in one-vessel disease was 13.0 ng/l (0.0–63.0); 2-vessel disease 15.0 ng/l (0.0–98.2); 3-vessel disease 15.0 ng/l (0.0–54.4), *P* = 0.94; and the median (range) hrCRP in one-vessel disease was 0.2 mg/dl (0-0.8); 2-vessel disease 0.4 mg/dl (0.1-0.8); 3-vessel disease 0.4 (0.1-0.7), *P* = 0.34), analysis of plasma sTREM-1 level by the number of stenosed/occluded coronary arteries yielded a positive correlation. Median (range) sTREM-1 levels increased from 282.7 pg/ml (255.6-429.4) in 1-vessel disease to 371.9 pg/ml (269.8-566.7) in 2-vessel disease and 577.2 pg/ml (451.3-1014.5) in 3-vessel disease (*P* < 0.001, Figure 3).

## 4. Discussion

To the best of our knowledge, our study is the first to demonstrate that higher plasma sTREM1-1 levels in patients presenting to the ED with recent-onset chest pain are significantly higher compared to HC, however, did not differentiate between ACS and NSCP (Tables 4 and 5, Figure 2). Furthermore, our results suggest that a higher plasma sTREM-1 level positively correlates with CAD severity, defined as a higher number of stenotic and/or occluded coronary arteries on coronary angiography, whereas neither serum troponin-T nor serum hsCRP levels correlate with angiography findings (Figure 2). Moreover, a higher plasma sTREM-1 level at admission to the ED was significantly associated with a higher risk of MI and stroke during a 6-month period (Table 3).

Inflammation plays a pivotal role in the formation of the atherosclerotic vascular plaque that underlies the pathological process of CAD [2–4, 6, 18, 19]. Focal subendothelial accumulation of apolipoprotein *β*-lipoproteins in the vascular wall induces an innate immune response dominated by monocytes/macrophages followed by an adaptive immune response [20].

Impaired resolution of the atherosclerotic vascular lesions leads to sustained, nonresolving, inflammation that promotes plaque progression and triggers acute thromboocclusive cardiovascular events [21]. As a result, monocytes differentiate into macrophages and lipid-laden foam cells, with further plaque development. Vulnerability to plaque formation is increased through cytokine, chemokine, and matrix metalloprotease production and through direct interactions with surrounding inflammatory and endothelial cells [22]. For example, the ox-LDL that accumulates in atherosclerotic plaque has been shown to “prime” monocytes for a subsequent increase in their inflammatory response to TLR2 and TLR4 activators [23]. TREM-1, a member of the immunoglobulin superfamily, is expressed on the cell surface of neutrophils and monocytes/macrophages in inflamed tissues [9, 10]. Following initial findings of elevated TREM-1 levels in infectious conditions such as sepsis [24], studies reported its upregulated membrane expression, as well as elevated blood and body fluid levels of sTREM-1, also in various noninfectious inflammatory conditions [25]. Upon TLR-4-mediated monocyte/macrophage activation, TREM-1 upregulation and signaling amplify the production of proinflammatory cytokines and chemokines, such as tumor necrosis factor alpha (TNF-*α*), interleukin- (IL-) 1*β*, IL-8, and IL-6 [9, 10], with concomitant shedding of its soluble form sTREM-1 [11]. Thus, sTREM-1 can be used as a marker of membrane TREM-1 upregulation as well as monocyte/macrophage activation. TREM-1 upregulation was demonstrated in murine and human atherosclerotic lesions *in situ*, and it was found to aggravate dyslipidemia-induced peripheral blood monocytosis [13]. Accordingly, one study showed that ox-LDL induced macrophage TREM-1 upregulation and foam cell formation, and silencing of TREM-1 expression by short hairpin interfering RNA inhibits macrophage lipid phagocytosis while reducing TNF-*α* and IL-6 production [8]. In humans, TREM-1 gene polymorphism has been associated with CAD [26]. Additionally, high plaque TREM-1 expression was found in symptomatic patients with carotid artery stenosis compared to asymptomatic patients, indicating that it plays a role in the stability of atherosclerotic plaques [27]. Others observed elevated plasma sTREM-1 levels in patients enrolled in a French registry of acute STEMI and NSTEMI (FAST-MI), with an adjusted hazard ratio (HR) of 2.22 for death at 2 years (*P* < 0.0001) [14]. Studies of *trem*^−/−^ mice as well as pharmacological inhibition of TREM-1 suggested that TREM-1 deficiency/inhibition significantly reduced atherosclerosis growth (in mice) and induced a plaque phenotype characterized by reduced macrophage infiltration and necrotic core size. TREM-1 deletion or blockade was associated with a significant and profound (up to 60%) reduction in the development of both early and advanced atherosclerosis. It also had a protective effect throughout the aorta (aortic sinus and ascending and descending aorta) [28], supporting the findings of a role for TREM-1 in monocyte infiltration and activation in plaques and foam cell formation. It has also been shown that TREM-1 apparently mediates ox-LDL-induced endothelial cell pyroptosis, a cell death process found in atherosclerosis, which is dependent on caspase-1 activation and IL-1*β* and IL-18 production [29–31].

Our results are in accordance with previous studies reporting elevated plasma sTREM-1 levels in acute MI [17, 22, 23]. Although plasma sTREM-1 levels above the 99th percentile of healthy control levels were positively associated with an increased risk of stroke on day 30 as well as increased risk of MI and stroke at month 6, they had no effect on the risk of MACE during the study 6 months follow-up period. By contrast, a recent prospective study of 838 patients with acute MI followed for 24 months observed that higher serum sTREM-1 levels were significantly associated with an increased risk of all-cause mortality as well as of MACE after adjusting for conventional risk factors [22, 23]. We suspect that the lack of a correlation with MACE in our study is explained by the short follow-up period (6 months vs. 24 months), and the fact that 3 (4.8%) patients in the NSCP group had a stroke following their admission to the ED as well as the zero-mortality rate in our cohort.

Our data suggest that elevated plasma sTREM-1 levels can be served as a biomarker of ACS in patients who present to the ED with a main complaint of chest pain, thereby expediting diagnosis and management. Moreover, given the correlation of plasma sTREM-1 level with the number of affected vessels, sTREM-1 may also serve as a biomarker for CAD severity in contrast to troponin-T level that did not correlate with the number of stenosed/occluded coronary arteries on angiography (Figure 2). The lack of association between plasma sTREM-1 levels and serum troponin-T levels implies that whereas troponin-T serves as a biomarker for myocardial injury, sTREM-1 is a biomarker for the sustained, low-grade inflammation involved in the pathological process of atherosclerotic plaque rupture that leads to ACS and MI.

We have previously shown that TLR-4 as well as TLR-9-induced macrophage activation results in a MMP-9-medited TREM-1 shedding [12]. Interestingly, increased MMP-9 expression can enhance extracellular matrix degradation and promote atherosclerotic plaque instability [32]. Moreover, MMP-9 is a strong independent predictor of atherosclerotic plaque instability in stable coronary heart disease and elevated MMP-9 levels are correlated with the size of the necrotic core of coronary atherosclerotic plaques [33]. Although we did not analyze plasma MMP-9 levels in our patients, these studies might suggest a possible link between MMP-9 and sTREM-1 levels in ACS that warrants further studies.

We have assessed the MACE 6 months following enrollment to the study and found that elevated plasma sTREM-1 levels at admission to the ED in the patients with ACS correlated with a higher risk for MI and/or stroke (Table 3).

Taken together with the correlation of plasma sTREM-1 with greater coronary disease involvement as defined by the number of affected arteries on angiography, our findings suggest that higher plasma sTREM-1 level is a biomarker for the severity of coronary disease as well as suggest that increased inflammatory response is a greater risk for atherosclerotic cardiovascular morbidity.

Our study is a case-control, longitudinal study with follow-up for 6 months; however, it was limited by the single-center setting, and relatively small sample size. These may explain the lack of a significant association between elevated plasma sTREM-1 level and either serum troponin or hsCRP levels. The zero-mortality rate precluded analysis of the potential association of plasma sTREM-1 level with risk of death or MACE.

## 5. Conclusion

Our study of elevated plasma sTREM1-1 levels in patients with ACS suggests that TREM-1 is involved in the inflammatory process underlying atherosclerosis and coronary plaque rupture, which lead to ACS. We propose that plasma sTREM-1 might serve as a biomarker of CAD severity as well as of the inflammation involved in the evolution of ACS.

## Figures and Tables

**Table 1:** Epidemiological characteristics of the study patients.

	Nonspecific chest pain (*n* = 62)	Acute coronary (*n* = 59)	Health control (*n* = 73)	*P* value
Plasma sTREM-1 pg/ml, median (i.q range)	402.2 (302.6-572.2)	429.4 (281.7-762.9)	218.9 (181.7-277.0)	<0.001
Age, mean (±SD)	54.1 (16.8)	58.4 (11.1)	42.6 (0.5)	<0.001
Males, *n* (%)	38 (61.3)	49 (83.1)	39 (53.4)	0.002
IHD, *n* (%)	9 (14.8)	30 (50.8)		<0.001
HTN, *n* (%)	21 (33.9)	39 (66.1)		0.001
Diabetes, *n* (%)	6 (9.7)	27 (45.8)		<0.001
Dyslipidemia, *n* (%)	25 (40.3)	45 (76.3)		<0.001
Statin therapy, *n* (%)	19 (30.6)	39 (66.1)	3 (4.1)	<0.001
Smoke status, *n* (%)				
Current	12 (20.3)	21 (35.6)		0.07
Past	14 (23.7)	13 (22.0)		
Never	33 (55.9)	25 (42.4)		
Family history of IHD, *n* (%)	17 (28.8)	31 (55.4)		0.01
Obesity, *n* (%)	10 (24.4)	29 (51.8)		0.01
BMI, mean (±SD)	27.1 (5.0)	28.6 (5.4)		0.21
Clinical presentation				
Pulmonary edema, *n* (%)	1 (1.6)	1 (1.7)		1.00
Cardiogenic shock, *n* (%)	0	4 (6.8)		0.04
STEMI, *n* (%)	0	22 (37.3)		
NSTEMI, *n* (%)	0	8 (13.6)		
Unstable/stable angina	0	29 (49.2)		
First troponin, median (i.q range)	0.0 (0.0-0.0)	15.0 (0.0-62.0)		<0.001
Second troponin, median (i.q range)	0.0 (0.0-21.7)	62.0 (0.0-1621.5)		<0.001
hrCRP, median (i.q range)	0.3 (0.1-0.6)	0.3 (0.2-0.7)		0.40
WBC	7.2 (2.1)	8.9 (3.0)		0.001
Creatinine	0.86 (0.22)	0.97 (0.32)	0.86 (0.20)	0.23
Heart score, median (i.q range)	2.0 (1.0-4.0)	5.0 (4.0-6.0)		<0.001
Grace score, median (i.q range)	0	100.0 (90.0-120.0)		
ED treatment				
Aspirin, *n* (%)	3 (4.8)	41 (69.5)		<0.001
Plavix/Brilinta/Effient, *n* (%)	0 (0.0)	18 (30.5)		<0.001
Heparin/LMWH, *n* (%)	0 (0.0)	21 (35.6)		<0.001
Nitrates, *n* (%)	1 (1.6)	8 (13.6)		0.01

i.q range: interquartile range.

**Table 2:** Diagnostic measures and clinical outcomes.

	Nonacute coronary (*n* = 62)	Acute coronary (*n* = 59)	*P* value
Admission unit			
ICCU, *n* (%)	0 (0.0)	33 (55.9)	<0.001
Ward, *n* (%)	15 (24.2)	24 (40.7)	
Home discharged, *n* (%)	47 (75.8)	2 (3.4)	
Cardiac catheterization			
Left main, *n* (%)	0 (0.0)	5 (8.5)	0.02
LAD, *n* (%)	0 (0.0)	26 (44.1)	<0.001
Diagonal, *n* (%)	0 (0.0)	12 (20.3)	<0.001
RCA, *n* (%)	0 (0.0)	26 (44.1)	<0.001
Marginal, *n* (%)	0 (0.0)	14 (23.7)	<0.001
Circumflex, *n* (%)	0 (0.0)	15 (25.4)	<0.001
PCI, *n* (%)	0 (0.0)	38 (64.4)	<0.001
CABG	0 (0.0)	4 (6.8)	0.05
Stroke day 30, *n* (%)	3 (4.8)	2 (3.4)	0.68
MI day 30, *n* (%)	0 (0.0)	2 (3.4)	0.14
Death day 30, *n* (%)	0 (0.0)	1 (1.7)	0.34
PCI day 30, *n* (%)	2 (3.2)	15 (25.9)	<0.001
MACE day 30^∗^	4 (6.5)	15 (25.4)	0.01
Stroke month 6, *n* (%)	3 (4.8)	2 (3.4)	0.68
MI month 6, *n* (%)	0 (0.0)	4 (6.8)	0.04
Death month 6, *n* (%)	0 (0.0)	1 (1.7)	0.34
PCI month 6, *n* (%)	2 (3.2)	17 (28.8)	<0.001
MACE month 6^∗^	4 (6.5)	17 (28.8)	0.01

^∗^CVA, MI, cardiac catheterization, or mortality.

**Table 3:** Clinical outcomes in patients with plasma sTREM‐1 < 99th percentile vs. patients with plasma sTREM‐1 > 99th percentiles.

	Plasma sTREM‐1 < 99th percentile^∗^ (*n* = 61)	Plasma sTREM‐1 > 99th percentiles^∗^ (*n* = 60)	*P* value
MACE day 30	7 (11.5)	12 (20.0)	0.21
CVA day 30	0 (0.0)	5 (8.3)	0.02
MI day 30	0 (0.0)	2 (3.3)	0.15
Mortality day 30	0 (0.0)	1 (1.7)	0.36
Cardiac catheterization day 30	7 (11.7)	10 (16.7)	0.60
MACE month 6	8 (13.1)	13 (21.7)	0.23
Stroke month 6	0 (0.0)	5 (8.3)	0.02
Recurrent MI month 6	0 (0.0)	4 (6.7)	0.04
Mortality month 6	0 (0.0)	1 (1.7)	0.36
Cardiac catheterization month 6	8 (13.1)	11 (18.3)	0.43
ACS	27 (44.3)	32 (53.3)	0.36
STEMI	15 (24.6)	7 (11.7)	0.10
NSTEMI	3 (4.9)	5 (8.3)	0.45
Single vessel disease	13 (48.1)	6 (19.4)	0.001
Two vessel disease	10 (37.0)	6 (19.4)	
Three vessel disease	4 (14.8)	19 (61.3)	

^∗^99th percentile of sTREM-1 in healthy control defined as 423 pg/ml.

**Table 4:** Logistic regression for developing ACS compared to noncardiac chest pain.

	*P* value	OR	95% CI
Age	0.11	1.02	0.99-1.06
Creatinine	0.98	0.98	0.17-5.66
Women	0.01	0.22	0.07-0.70
First troponin	0.02	1.02	1.01-1.05
sTREM-1/100	0.01	1.29	1.07-1.54

**Table 5:** Logistic regression for developing ACS compared to noncardiac chest pain.

	*P* value	OR	95% CI
Age	0.11	1.02	0.99-1.06
Creatinine	0.98	0.98	0.17-5.66
Women	0.01	0.22	0.07-0.70
First troponin	0.02	1.02	1.01-1.05
sTREM-1/100	0.01	1.29	1.07-1.54

## Data Availability

Data will be available to request.
